# Timing of outdoor light exposure is associated with sleep-wake consolidation in community-dwelling older men

**DOI:** 10.3389/frsle.2023.1268379

**Published:** 2023-10-19

**Authors:** Renske Lok, Sonia Ancoli-Israel, Kristine E. Ensrud, Susan Redline, Katie L. Stone, Jamie M. Zeitzer

**Affiliations:** ^1^Department of Psychiatry and Behavioral Sciences, Stanford University, Stanford, CA, United States; ^2^Department of Psychiatry, University of California, San Diego, San Diego, CA, United States; ^3^Division of Epidemiology and Community Health and Department of Medicine, University of Minnesota, Minneapolis, MN, United States; ^4^Department of Medicine and Neurology, Brigham and Women's Hospital, Harvard Medical School, Boston, MA, United States; ^5^Department of Epidemiology, Harvard TH Chan School of Public Health, Boston, MA, United States; ^6^California Pacific Medical Center Research Institute, San Francisco, CA, United States; ^7^Mental Illness Research Education and Clinical Center, VA Palo Alto Health Care System, Palo Alto, CA, United States

**Keywords:** light, sleep-wake fragmentation, receiver operating characteristic curves, circadian, amplitude, aging

## Abstract

**Introduction:**

A consolidated sleep-wake pattern is essential for maintaining healthy cognition in older individuals, but many suffer from sleep fragmentation that exacerbates age-related cognitive decline and worsens overall mental and physical health. Timed light exposure (light therapy) has been explored as a countermeasure, but mixed results have been obtained. To determine whether the timing of light exposure is important for sleep-wake consolidation, we analyzed the natural light diets of a cohort of community-dwelling older men.

**Methods:**

The degree of sleep-wake fragmentation and light exposure patterns were obtained over a week using wrist actigraphy. Correlations between fragmentation, light patterns, and various physical and mental health measures were examined (*n* = 877).

**Results:**

Our findings revealed that higher sleep-wake fragmentation correlated with poorer physical and mental health and reduced cognition. Moreover, reduced daytime light exposure was associated with increased sleep-wake fragmentation. Interestingly, morning and evening light exposure (>1,000 lux) were not useful in distinguishing between low and high sleep-wake fragmentation scores, while increased afternoon light exposure showed much better discrimination. Specifically, optimal discrimination between low and high fragmentation occurred 6.7 h after habitual sleep offset. This suggests that afternoon light therapy might be more efficient in consolidating sleep and wake in older adults, particularly in those with low-amplitude circadian rhythms.

**Discussion:**

This study highlights the significance of properly-timed light exposure in promoting consolidated sleep and cognitive health among older individuals. Tailored light-based strategies may have the potential to enhance physical, mental, and cognitive well-being in the aging population.

## 1. Introduction

Consolidated sleep is crucial to maintaining physical (Rosseland et al., 2018) and mental wellbeing (Yaffe et al., 2014). However, as individuals age, their ability to maintain consolidated periods of sleep tends to decline, resulting in fragmented sleep-wake patterns and increased daytime sleepiness (Mander et al., 2017). The underlying mechanisms behind these age-related sleep changes are not fully understood. It has been postulated, however, that the degeneration of the circadian clock in the hypothalamic suprachiasmatic nuclei (SCN) may play a pivotal role in the disruption of sleep-wake patterns (van Someren et al., 1997).

The SCN regulates daily sleep-wake cycles (Daan et al., 1984; Borbély et al., 2016) and synchronizes internal rhythms with the external light-dark cycle, primarily through direct input from the retina to the SCN (Provencio et al., 2000; Berson et al., 2002; Hattar et al., 2002). The impact of light on circadian rhythms varies both in terms of the timing of the light exposure as well as intensity (Jewett et al., 1994; St Hilaire et al., 2012). Super healthy older adults are less sensitive to light than younger adults, suggesting that older individuals require more light exposure to achieve the same benefits experienced by younger individuals (Duffy et al., 2007). Common age-related ocular conditions, such as cataracts and lens yellowing, further diminish circadian light sensitivity by reducing the amount of light reaching the retina (Barker, 1991; Charman, 2003; Turner and Mainster, 2008). Behavioral changes can also contribute to reduced light exposure as older individuals often reside in poorly lit environments and have limited exposure to natural daylight, particularly during winter (Bakker et al., 2004; Flores-Villa et al., 2020). It has been hypothesized that this type of insufficient light exposure in older adults can lead to disrupted circadian rhythms, which contribute to greater sleep-wake fragmentation (Ancoli-Israel et al., 2002).

One commonly explored countermeasure to sleep fragmentation in older adults is intentional increased exposure to bright light (light therapy). However, light therapy studies have reported mixed results, with both reduced (Hozumi et al., 1990; Okawa et al., 1991; Satlin et al., 1992; Lovell et al., 1995; van Someren et al., 1997; Van Someren, 2000) and unchanged sleep-wake fragmentation reported (Colenda et al., 1997; Friedman et al., 2012; De Rui et al., 2015; Mitolo et al., 2018). These mixed results may have to do with the varied methodologies used in these studies, with the timing, intensity, and duration of light exposure often being different across investigations. As the circadian system can have diametrically opposed responses to light depending on its timing (Jewett et al., 1994), when the light therapy occurs appears to be an important factor in predicting responses, though it has been argued that light therapy timing should be a matter of convenience rather than based on the underlying cause of the disorder (Sloane et al., 2008). Traditionally, morning light therapy has been considered a primary therapeutic approach to consolidate sleep and wake, assuming poor synchronization of the circadian clock is the primary cause of increased sleep-wake fragmentation (Satlin et al., 1995). Several studies, however, suggest that heightened sleep-wake fragmentation might be a consequence of diminished circadian amplitude rather than poor synchronization (Welsh et al., 1986; Huang et al., 2002). While morning light therapy effectively addresses synchronization-related issues in the circadian clock, it does not elicit substantial changes in its amplitude (Jewett et al., 1994). Laboratory studies indicate that the most robust changes to circadian amplitude occur following exposure to light ~10 hours after the minimum of core body temperature (i.e., around 4:00 p.m. in an individual habitually awakening at 8 a.m.) (Jewett et al., 1994), a time at which there is minimal impact of light on the timing of the circadian clock (St Hilaire et al., 2012). Thus, bright light in this late afternoon time could specifically enhance circadian amplitude, leading to an increase in sleep-wake consolidation.

To initially examine the hypothesis that bright light in the late afternoon time may specifically enhance circadian amplitude, leading to an increase in sleep-wake consolidation, we examined the natural light diet of older community-dwelling men and the association of these light patterns with sleep-wake consolidation. The impact of sleep-wake fragmentation on physical and mental health was also considered.

## 2. Materials and methods

### 2.1. Sample

Original data were collected during the Osteoporotic Fractures in Men study (MrOS), which took place from March 2000 to April 2002 (*n* = 5,994, community-dwelling men aged 65 years or older). This enrollment occurred at six clinical centers located in different regions of the United States. Before participating, written consent was obtained from all participants, adhering to the principles outlined in the Declaration of Helsinki. The comprehensive details of the study methodology have been previously published (Blank et al., 2005; Orwoll et al., 2005).

The ancillary “MrOS Sleep Study” recruited 3,135 participants from the original cohort (known as “Sleep Visit 1”) and focused on conducting a comprehensive assessment of sleep patterns. All active participants from the MrOS Sleep Study who had objective sleep data during Sleep Visit 1 were eligible to take part in Sleep Visit 2, which occurred between November 2009 and March 2012 (*n* = 1055). During Sleep Visit 2, light exposure measurements were added to the battery of data collected. For the present analyses, we used light and activity data obtained through actigraphy, as well as relevant covariate data from Sleep Visit 2.

### 2.2. Actigraphy—Sleep-wake fragmentation

The Actiwatch 2 (Philips Respironics Inc, Murrysville, PA), was used to measure light and movement (accelerometry) signals. Actigraphs were worn on the nondominant wrist for at least five consecutive 24-h periods, except when bathing or during water sports. Epochs in which the actigraph was removed were not used in the calculation. Accelerometry signals were analyzed using non-parametric analyses. To quantify sleep-wake fragmentation, intradaily variability [IV, nparAct (Blume et al., 2016)] was calculated, defined as $IV\;=\;\frac{n\overset{n}{\underset{i\;=\;2}{\sum }}{\left(X_{i}-X_{i-1}\right)}^{2}}{\left(n-1\right)\overset{n}{\underset{i\;=\;1}{\sum }}{\left(X_{i}-\bar{X}\right)}^{2}}$, such that *n* is the total number of hours in the data collection, *x* is the hourly average, and *x*_*i*_ is the average across all hours. IV values range from 0 to 2, with higher values representing greater sleep-wake fragmentation. Habitual sleep onset and offset were derived from accelerometry signals, such that sleep onset was calculated as the first minute of a block of 10 min with 9 or more minutes scored as immobile (activity count <4), averaged over all nights, and habitual sleep offset was calculated as the last minute of the last block of 10 min with 9 or more minutes scored as immobile, averaged over all nights. We used the longest nocturnal period as the main sleep episode.

### 2.3. Actigraphy—Light exposure profiles

The actigraph used in the study was equipped with an ambient light sensor capable of gathering data on light illuminance, measured in lux with a silicon photodiode (wavelength range: 400–900 nanometers) and could detect illuminance levels up to 100,000 lux, with a 10% accuracy at 3,000 lux. Data were stored as average light in 1-minute epochs. Light outputs were quantified in three ways: (1) average white light exposure per minute; (2) mean of sum of total white light exposure; (3) minutes spent in >1,000 lux (time above threshold, TALT). Light values below 1 lux were substituted with 1 in the calculation, assuming that the light sensor's resolution was not <1 lux. All output measures were calculated between habitual sleep offset and onset, and were averaged over all recording days. To explore if the effects of outside light exposure depend on the time of day, for each individual, TALT was calculated in 1-h bins, starting from habitual sleep offset. To explore the spectral diet during the active period, light exposure levels were characterized up to 14 h after habitual sleep offset.

### 2.4. Exploratory differences

To better understand the relationship between sleep-wake fragmentation, physical and mental well-being, the correlation between IV and a variety of measures was investigated, including overall health [Physical Activity Scale for the Elderly (PASE), including total, and individual items (Washburn et al., 1993), Physical Component Summary (PCS) (Ware et al., 1998), Instrumental Activities of Daily Living (IADL)] (Pincus et al., 1983; Fitti and Kovar, 1986), overall mental health [Mental Component Summary (MCS) from self-rated health and health-related quality of life (SF-12)] (Ware et al., 1998), depression [geriatric depression scale (GDS)] (Yesavage et al., 1982), and cognition [Modified Mini-Mental State, (3 MS)] (El, 1987).

### 2.5. Sleep-wake fragmentation and light exposure profiles

**Figure 1:**
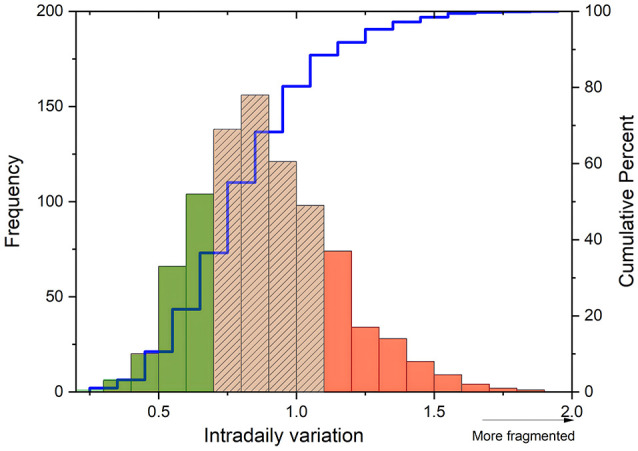
Categorization of sleep-wake fragmentation. Frequency distribution (y-axis) of Intradaily Variation scores (x-axis) and its categorization based on the cumulative percentage (right y-axis) of sleep-wake fragmentation levels. IV scores were ranked and categorized into low (green), medium (striped), or high (red) degrees of sleep-wake fragmentation based on this distribution.

The correlational relationship between light output measures (average light exposure, mean of sum of total light exposure, TALT) and degree of sleep-wake fragmentation was explored first. Afterward, IV scores were ranked and divided into high, medium, or low levels of sleep-wake fragmentation categories based on the distribution of IV scores (Figure 1). Low or high sleep-wake fragmentation was defined as IV scores <0.67 (*n* = 175) or >1.1 (*n* = 176), respectively (Figure 1). To assess the ability of light information to distinguish high from low sleep-wake fragmentation, Receiver Operating Characteristic (ROC) curves were constructed for each hour, with the degree of sleep-wake fragmentation as a dependent and TALT as an explanatory parameter. To assess at which time daylight information is best at discriminating high from low sleep-wake fragmentation, Youden's J statistic, defined as J = sensitivity + specificity−1, was constructed for each hour, with 1 indicating perfect classification and 0 indicating no discrimination between high and low degree of sleep-wake fragmentation.

### 2.6. Statistics

Linear model fitting was performed in R (R Core Team, version: 4.1.2), using the most recent shell of RStudio (version: 2021.09) and the “lme4” R-package for mixed-effects modeling. Independent models were constructed to calculate the correlation between sleep-wake fragmentation and physical and mental health, and cognition. Dependent variables were PASE, PCS, IADL, MCS, GDS, and 3MS, while IV scores were added as fixed effects. A p-value of 0.05 was maintained for all statistical analyses. Effect sizes were determined by calculating eta squared (using package “lsr”), such that η^2^ between 0.01-−0.06 is a small effect size, η^2^ between 0.06 and 0.14 is a medium effect size and η^2^ ≈ 0.14 or larger is considered a large effect size.

To determine the correlation between sleep-wake fragmentation and light parameters, an exponential decay formula was fitted to the data $y\;=\;A_{0}e^{-kt}$, with *A*_0_ = initial amount, *e* is Euler's constant, and *k* is a negative constant that determines the rate (percentage) of decay.

To generate ROC curves (with sleep-wake fragmentation as the dependent variable and TALT at various times of day as independent variables) and Youden's J statistic, package “ROCR” was used. To predict the optimal timing, a Gaussian fit for peak detection was constructed as follows: f = ($\frac{1}{\sigma^{*}{\sqrt{2\pi }}^{*}\exp(-({\left(x-u\right)}^{2}/2\sigma^{2}}$), with *f* representing the probability density at a given point *x*, σ the standard deviation, μ the mean, and π the mathematical constant pi. All plots and curve fits were constructed in OriginPro 2021b (version: 9.8.5.212, Origin Lab, Northampton MA). Data are presented as median [2^nd^ and 3^rd^ quartile] unless otherwise specified.

## 3. Results

### 3.1. Description of sample

**Figure 2:**
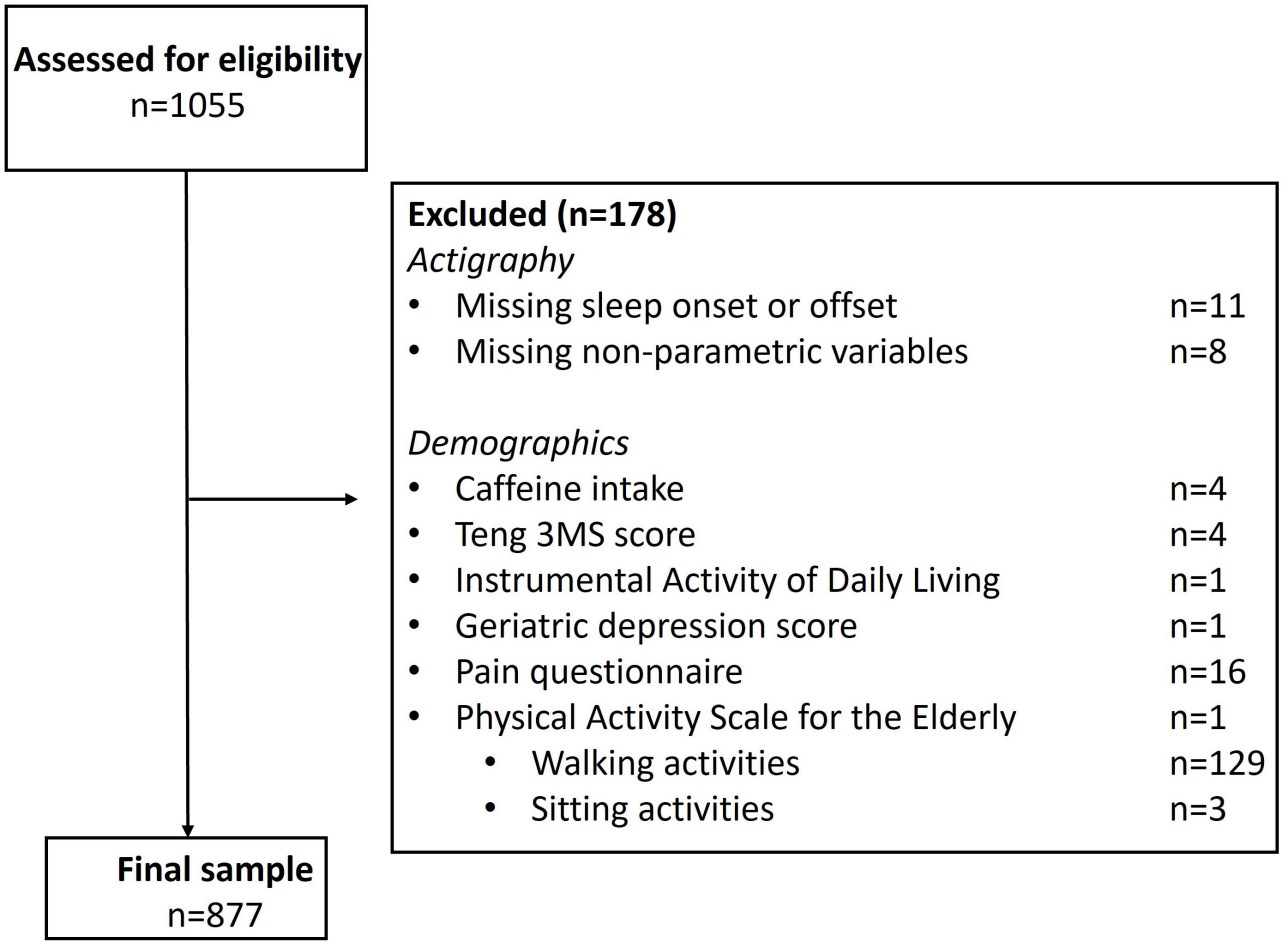
Participant inclusion and exclusion criteria. All participants in Sleep Visit 2 (*n* = 1055) were assessed for eligibility, with 178 excluded, resulting in 877 included individuals. Note, on the PASE, only one participant was missing this questionnaire in its entirety; 132 participants were missing individual subscale scores, as noted, and excluded.

The initial sample had 1,055 older adult males. Following exclusions for missing data, we had a sample of 877 adults (Figure 2).

The sample consisted entirely of male participants, with a median age of 80 [77–81] years and primarily white ethnicity (87%). Their self-reported caffeine intake was 220 mg/d [48–247 mg/d]. Their cognitive functioning was assessed to be good, with a median 3MS score of 94.00 [90.00–92.48]. There were 20 individuals (2.3%) in whom the 3MS score was <77, the threshold for mild cognitive impairment. Activities of daily living were not reduced, as indicated by a score of 0 impairments in performance in 72.5% of the study sample. Most individuals did not exhibit signs of depression, with 91.4% scoring below the depression cutoff [GDS<5 (Yesavage, 1988)]. This finding was also consistent with the participants' MCS scores (57.8 [53.6–60.2]). In terms of physical activity, most individuals were relatively active, with a median PASE score of 125.1 [81.46-169.2], and they reported mild physical disabilities, as indicated by a median PCS score of 51.04 [40.06-56.58]. Most participants (64.0%) did not experience any bodily pain in the past month, while some reported pain less than once a week (13.1%), once or twice a week (9.42%), or three or more times a week (13.3%). The intradaily variability (IV) values ranged from 0.31 to 1.89, with a median score of 0.87 [0.72–1.05] (Figure 1). Participants initiated sleep at a median time of 23:30 [22:37–23:22 h], they awakened at a median time of 7:02 [06:24–07:40 h], and were exposed to a median of 35 min [10.00–84.50 min] of bright (>1,000 lux) light per day. Data were collected during winter (*n* = 217), spring (*n* = 207, summer (*n* = 209), and fall (*n* = 244).

### 3.2. Associations with sleep-wake fragmentation

Greater sleep-wake fragmentation correlated with a more sedentary lifestyle (PASE, F_2,875_ = 90.49, *p* < 0.0001, η^2^ = 0.094) and more impairments in performing activities of daily living (IADL, F_2,875_ = 31.86, *p* < 0.0001, η^2^ = 0.035). Higher sleep-wake fragmentation was also characterized by lower cognitive scores (3MS, F_2,8751_ = 4.215, *p* = 0.040, η^2^ = 0.005), worse mental health (SF-12 MCS, F_2,875_ = 7.226, *p* = 0.007, η^2^ = 0.007) and higher depression scores (GDS, F_2,875_ = 11.44, *p* < 0.0001, η^2^ = 0.013). Sleep-wake fragmentation was not associated with frequency of experienced bodily pain (F_2,875_ = 0.007, *p* = 0.934) or average daily caffeine intake (F_2,875_ = 0.98, *p* = 0.322). As the relationship between sleep-wake fragmentation and PASE score exhibited an effect size larger than medium (η^2^ > 0.06), this relationship was further explored.

### 3.3. PASE scores and sleep-wake fragmentation

There were significant associations between IV scores and specific questions on the PASE including outdoor gardening (F_4,872_ = 22.04, *p* < 0.0001, η^2^ = 0.027) and hours of walking outside (F_4,872_ = 25.60, *p* < 0.0001, η^2^ = 0.025). Other questions referring to outdoor activities, including lawn care (F_4,872_ = 3.082, *p* = 0.079) and how often individuals walked (F_4,872_ = 2.130, *p* = 0.145), did not significantly correlate with IV. Together, outdoor gardening and hours of walking outside explained ~7.66% of the variance in sleep-wake fragmentation, suggesting that being outside may be associated with lower sleep-wake fragmentation. We, therefore, explored the relationship between light exposure and sleep-wake fragmentation.

### 3.4. Light effects on sleep-wake fragmentation

**Figure 3:**
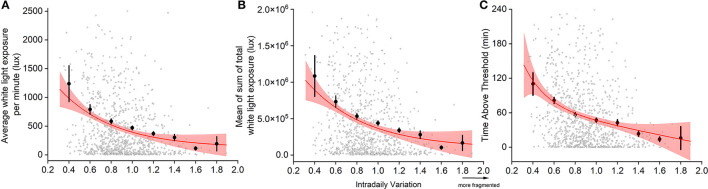
Relationship between sleep-wake fragmentation and light exposure: average white light exposure per minute out of bed **(A)**, mean of sum of total white light exposure out of bed **(B)**, and minutes spent in light >1,000 lux (TALT) **(C)**. Individual data (open circles), binned averages (closed circles), and the fitted curve and 95% confidence intervals (red lines and shaded area) are presented. Due to outliers skewing the graph, we omitted specific data from visualization, but not statistical analysis. Of 877 datapoints, 1.5% of data presenting mean light (cutoff: 2,500 lux), 2.4% of the sum of light (cutoff: 200,000 lux), and 1.4% of TALT (cutoff: 240 min) were omitted.

There was a significant correlation between sleep-wake fragmentation (IV scores) and both the average amount of daytime light exposure (F_2,875_ = 60.354, *p* < 0.0001, *R*^2^ = 0.065, Figure 3A) and the mean sum of daytime light exposure (F_2,874_ = 32.55, *p* < 0.0001, *R*^2^ = 0.069, Figure 3B), such that more sleep-wake fragmentation was correlated to lower levels of light exposure. The time spent above threshold (F_2,875_ = 68.076, *p* < 0.0001, *R*^2^ = 0.072) showed the strongest correlation to sleep-wake fragmentation, suggesting that spending less time in light >1,000 lux (generally considered being outdoors) was correlated with higher degrees of sleep-wake fragmentation (Figure 3C).

**Figure 4:**
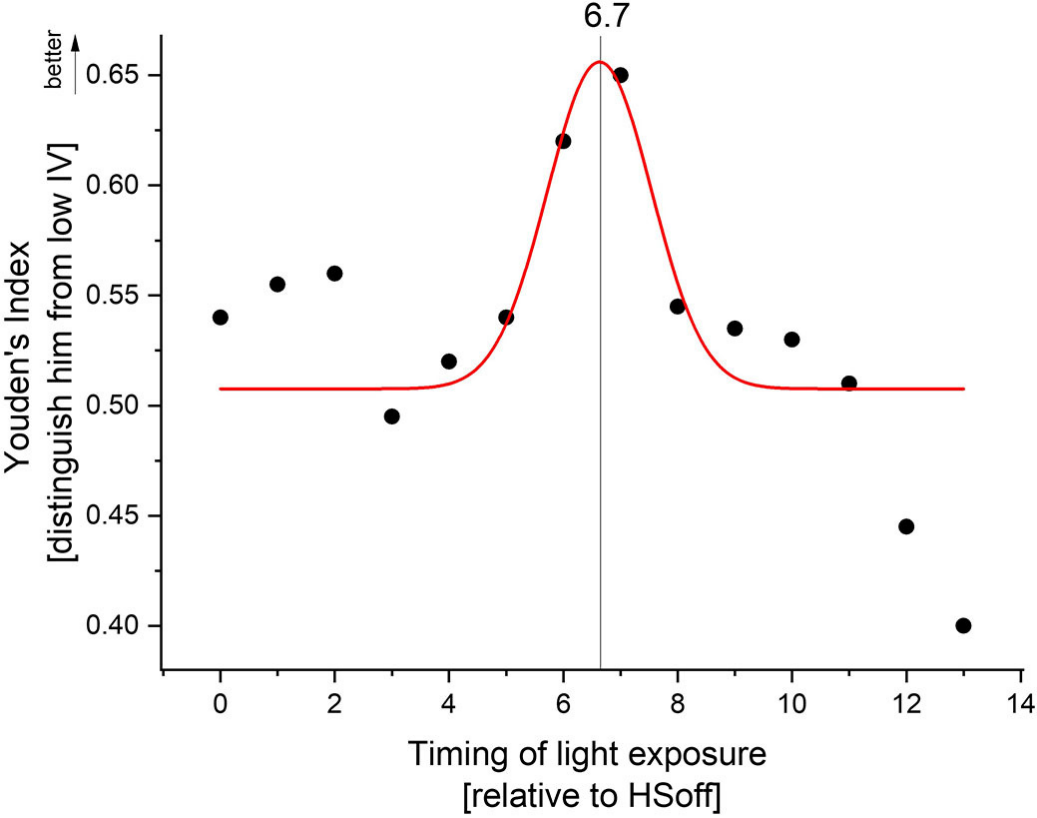
Receiver operating characteristic (ROC) curves, in which sensitivity of discriminating between high and low sleep-wake fragmentation based on high or low time above threshold was plotted against 1-specificity. Youden's J statistic, which captures the performance of the ROC, was calculated for each hour after HSoff. Gaussian peak fitting indicated optimal discrimination between high and low sleep-wake fragmentation 6.7 h after habitual sleep offset.

ROC curves were generated to determine to what degree the time spent above >1,000 lux per hour can distinguish high from low sleep-wake fragmentation. Based upon light exposure profiles, there was good discrimination (Youden's J ≥ 0.5) between high and low sleep-wake fragmentation using TALT, indicating that light exposure patterns are relatively sensitive in discriminating high from low sleep-wake fragmentation (Figure 4). The Gaussian fit for peak detection was significant (F_3,14_ = 3.96, *p* = 0.04, *R*^2^ = 0.18) and suggested that the optimal discrimination between low and high sleep-wake fragmentation occurs 6.7 h (6 h and 42 min) after habitual sleep offset, indicating that exposure to outdoor light at this time of day (e.g., 1:42 p.m. in an individual awakening at 7:00 a.m.) is associated with greater sleep-wake consolidation.

## 4. Discussion

Our data indicate that in a community-dwelling cohort of older men, *ad libitum* exposure to bright light during the early afternoon is associated with a greater consolidation of sleep and wake. Our data are consistent with the hypothesis that such light exposure may increase the amplitude of the circadian clock, which is responsible for the more desirable pattern of increased sleep-wake consolidation. We also found that older men with a higher degree of sleep-wake fragmentation had a more sedentary lifestyle, greater impairments in performing instrumental activities of daily living, worse physical and mental health, and lower cognitive performance. As this is a cross-sectional, correlational study, causality cannot be inferred or established, but the data are nevertheless supportive of the premise that targeting light exposure at this time of day may be beneficial to older individuals with fragmented sleep.

Sleep-wake fragmentation, characterized by frequent awakenings or interrupted sleep throughout the night and increased daytime sleepiness, is common in older individuals (Luik et al., 2013). Consequences of disrupted sleep architecture and quality profoundly affect physical and mental health (Yaffe et al., 2014; Rosseland et al., 2018). Previous trials of light therapy as a countermeasure to sleep-wake fragmentation have been inconclusive (Mitolo et al., 2018). Our data indicate that some of the variability in these trials may be due to the light being mistimed as the light therapy was conducted in the early morning or late evening, times during which the impact of light on circadian phase is strong but its impact on circadian amplitude is weak (Jewett et al., 1994). A direct randomized trial of proactively assigning light therapy in the midafternoon compared with morning is warranted to answer this question. Future interventional studies should also examine whether the entire hour needs to be spent in bright (>1,000 lux) light or whether shorter durations are equally as useful.

Sleep plays a crucial role in cognitive processes such as attention (Stepanski et al., 1987), memory, and learning (Bonnet, 1985; Djonlagic et al., 2012), and fragmentation significantly impairs such functioning. Sleep-wake fragmentation is also linked to an increased risk of physical disorders, including obesity (Wang et al., 2014; Zhao et al., 2021), metabolic dysregulation (Stamatakis and Punjabi, 2010), and cardiovascular problems like hypertension and heart disease (Stamatakis and Punjabi, 2010). Sleep and mental health have a bidirectional relationship, such that sleep-wake fragmentation contributes to developing and worsening mental health disorders through increasing stress levels (Tartar et al., 2009), anxiety (Grubac et al., 2019), and depression (Pesonen et al., 2019), while mental health disorders can also induce sleep-wake fragmentation (Lucchesi et al., 2005). Sleep-wake fragmentation is particularly common in older individuals with neurodegenerative disorders (Deuschle et al., 2014; Liguori et al., 2016) and the co-occurrence with sleep-wake fragmentation is thought to exacerbate both cognitive and behavioral difficulties in these individuals (Moran et al., 2005; Guarnieri et al., 2012; André et al., 2019). Even though there were very few individuals with mild cognitive impairment in our dataset, our data are consistent with the hypothesis that sleep-wake fragmentation is associated with lower cognitive performance and reduced physical and mental health. Given the broad impacts of sleep-wake fragmentation on physical and mental health, future research is needed to evaluate whether targeted light therapy results in broad changes in a number of age-related domains that may improve quality of life.

Several other investigations have shown that insufficient light exposure is associated with sleep-wake fragmentation in older individuals (Swaab et al., 1985; Ancoli-Israel et al., 1989; Shochat et al., 2000; Weinert, 2000; Nakamura et al., 2011). Older individuals may be particularly susceptible to this given an age-related decline in light sensitivity (Barker, 1991; Charman, 2003; Turner and Mainster, 2008) and limited exposure to natural daylight, particularly during winter (Bakker et al., 2004; Flores-Villa et al., 2020). Participants were exposed to a median of 35 min [10.00–84.50 min] of light >1,000 lux per day, which is either comparable to what has been reported in other community-dwelling populations (Flores-Villa et al., 2020) or slightly less (Campbell et al., 1988). However, the duration of high-intensity light exposure reported is much longer than those in institutionalized individuals, who spend a median of 9 min above 1,000 lux (Ancoli-Israel et al., 1997). Insufficient light exposure, leading to diminished circadian rhythms (Swaab et al., 1985; Weinert, 2000; Nakamura et al., 2011), is a likely cause of sleep-wake rhythm fragmentation (Ancoli-Israel et al., 1989; Shochat et al., 2000). Previous strategies involve increasing illumination throughout the day using either artificial ambient light or light therapy devices (Hozumi et al., 1990; Okawa et al., 1991; Satlin et al., 1992; Lovell et al., 1995; van Someren et al., 1997; Van Someren, 2000; Friedman et al., 2012). While passive strategies of increasing ambient illumination may be useful, they are limited in that the intensities to which individuals are exposed are often much lower than outdoor light levels. It is unclear whether such elevated (i.e., >1,000 lux) intensities are needed to change circadian amplitude and subsequent sleep-wake consolidation under ambulatory conditions. Furthermore, the duration of such an exposure is also unclear. These will be important characteristics of light to delineate going forward to make an evidence-based intervention.

The current study used actigraphy signals and actigraph-acquired light information for data analyses. Actigraphy provides a non-invasive method for assessing sleep-wake patterns in real-world settings (Ancoli-Israel et al., 2003), but it has certain limitations, including reduced sleep-detection accuracy in individuals with fragmented sleep (Tryon, 2004; Zeitzer et al., 2020) and accurately measuring light information from the wrist, which differs from light entering the eye (Yoshimura et al., 2022). Our non-parametric approach does not depend on the precise identification of “sleep” and “wake,” but rather on the distribution of activity levels. Further investigation into the impact of enhanced daytime light exposure on polysomnographically-determined sleep stages and microarchitecture is also important going forward. Regarding the wrist-based light measurements, while these are unable to capture subtle variation in ocular light exposure accurately, they are sufficient to detect when ambient illumination is above 1,000 lux, which typically only happens when one is outside or close to a window (Joyce et al., 2020). The protocol did not mandate the light meter to remain uncovered, raising the possibility that misclassification may have been introduced as a result of clothing covering the light meter, particularly during colder weather. We were unable to consider the potential impact of seasonal variations in weather that could have influenced the duration of exposure to outdoor light. This would be important to include in future investigations. Furthermore, the cross-sectional nature of the study design infers the possibility of assessing temporal causality. At best, we can determine associations between greater sleep-wake fragmentation, light, and health outcomes, in sample of predominantly non-Hispanic White men. Lastly, greater sleep-wake fragmentation is potentially associated with light exposure at times of day when darkness would be beneficial for the circadian system (i.e., night). The occurrence of reversed causality i.e., light exposure at unusual times of day as a cause of greater sleep-wake fragmentation, cannot be excluded.

In conclusion, this study highlights the potential importance of afternoon light exposure in consolidating sleep-wake patterns in older individuals. While morning or evening light therapy may be suitable for some older individuals, particularly those for whom the timing of the circadian system is an underlying issue, afternoon light therapy may be a promising alternative for those with underlying circadian amplitude problems. By increasing circadian amplitude without affecting clock phase, afternoon light exposure may offer a more effective approach to consolidating sleep-wake patterns in this population. Development of algorithms to determine the etiology of sleep-wake fragmentation will be crucial in the use of precision light therapy as a treatment modality. This would represent a paradigm shift in the way we approach light-based interventions, acknowledging the significance of timing in achieving optimal therapeutic outcomes. Ultimately, our research contributes to the emerging field of precision sleep medicine and underscores the importance of tailoring treatment strategies to individual underlying circadian etiology to optimize sleep quality and cognitive health in aging populations.

## Data availability statement

Publicly available datasets were analyzed in this study. This data can be found here: https://mrosonline.ucsf.edu.

## Author contributions

RL: Conceptualization, Data curation, Formal analysis, Investigation, Methodology, Visualization, Writing—original draft. SA-I: Writing—review and editing. KE: Writing—review and editing. SR: Writing—review and editing. KS: Writing—review and editing. JZ: Conceptualization, Supervision, Visualization, Writing—review and editing.
